# IMPACT OF STATE-LEVEL FACTORS ON CONSUMER-REPORTED UNMET NEEDS IN HOME AND COMMUNITY-BASED SERVICES

**DOI:** 10.1093/geroni/igae098.1943

**Published:** 2024-12-31

**Authors:** Romil Parikh, Tetyana Shippee, Benjamin Langworthy, Zheng Wang, Eric Jutkowitz, Stephanie Giordano

**Affiliations:** University of Minnesota, Minneapolis, Minnesota, United States; University of Minnesota School Of Public Health, Minneapolis, Minnesota, United States; University of Minnesota, Minneapolis, Minnesota, United States; University of Minnesota School of Public Health, Minneapolis, Minnesota, United States; Brown University, Brown University, Rhode Island, United States; Human Services Research Institute, Cambridge, Massachusetts, United States

## Abstract

In the value-based purchasing framework, provider-specific quality indicators are often compared with national average estimates to measure performance. Hence, it is important to elucidate the impact of state-level administrative factors on consumer-level quality indicators for their national use. Therefore, we evaluated associations of state-level factors (from publicly reported data) with consumer-reported service-specific unmet needs for six commonly used home and community-based services (HCBS) from the NCI-AD data (2015-2019, n=23,333 community-dwelling older respondents; age, ≥65 years). We fit a mixed-effects logistic regression model for unmet needs in each of six HCBS; adjusting for state ID random effect, consumer-level health and demographic fixed effects, and four state-level fixed effects including HCBS spending relative to institutional care spending, percent managed care population, HCBS spending per client, and Medicaid expansion. To evaluate whether addition of these 4 state-level fixed effects improved model fit, we evaluated improvement in the Akaike Information Criterion, for each of six HCBS. A 10% increase in the proportion of managed care population in state was significantly associated with greater odds of consumer-reported unmet needs in personal care (Odds Ratio [OR], 1.37, p<0.01), homemaker services (OR, 1.74, p<0.001), and transportation services (OR, 1.22. p<0.05). There were no significant associations between other state-level factors and service-specific unmet needs. Addition of the four state-level factors improved model fit for personal care, homemaker, meal delivery, and transportation services, and worsened model fit for adult day and caregiver-respite services. State-level factors likely have very little impact on consumer-reported unmet needs as an HCBS quality indicator.

